# Anti-EGFR-Coated Gold Nanoparticles In Vitro Carry 5-Fluorouracil to Colorectal Cancer Cells

**DOI:** 10.3390/ma13020375

**Published:** 2020-01-14

**Authors:** Raquel B. Liszbinski, Graziela G. Romagnoli, Carolina M. Gorgulho, Caroline R. Basso, Valber A. Pedrosa, Ramon Kaneno

**Affiliations:** 1 Department of Microbiology and Immunology, Institute of Bioscience, UNESP, Botucatu, SP 18618-000, Brazil; raquelb.biomed@gmail.com (R.B.L.); graziela.romagnoli@gmail.com (G.G.R.); Carolina.gorgulho@gmail.com (C.M.G.); 2 Department of Chemistry and Biochemistry, Institute of Bioscience, UNESP, Botucatu, SP 18618-000, Brazil; caroll.rodrigues09@gmail.com (C.R.B.); valber.pedrosa@unesp.br (V.A.P.)

**Keywords:** colorectal cancer, cancer therapy, drug delivery, gold nanoparticles, nanocarriers, monoclonal antibodies

## Abstract

The aim of the current study is to present a strategy to improve the efficiency of 5-fluorouracil (5-FU), which is widely used as antineoplastic agent against solid tumors-based on the use of gold nanocarriers to overcome the resistance of colorectal cancer cells. 5-FU was loaded on gold nanoparticles (AuNP) coated with anti-EGFR antibodies in order to target them towards colorectal cancer cells that overexpress epidermal growth factor receptors (EGFR). Physicochemical characterization has shown that AuNP size was approximately 20 nm and that AuNP functionalization led to spherical nanoparticles. Flow cytometry allowed observing that some compounds synthesized by our research group have induced apoptosis/necrosis and impaired the proliferation of colon cancer cell lines ‘HCT-116′ and ‘HT-29′. The antibody/drug combination in AuNP (AuNP 5FU EGFR) has improved the apoptosis rate and impaired cell proliferation in both cell lines, regardless of the exposure time. Overall, these results have shown that AuNP functionalization with monoclonal antibodies focused on delivering 5-FU to tumor cells is an exciting strategy against colorectal cancer.

## 1. Introduction

Colorectal cancer (CRC) is highly incident, as well as the third most significant cause of death among men and women [1]. Unfortunately, CRC metastasis and relapse are often observed, and it hinders patients’ complete remission [2]. A range of chemotherapeutic agents is often used to treat CRC, either alone or in combination with other drugs, in order to prolong patients’ life [3,4,5]. The 5-fluorouracil (5-FU) is the most common chemotherapeutic drug [6]. It is a pyrimidine-analog antineoplastic antimetabolite that interferes in DNA synthesis by blocking deoxyuridylic acid conversion into thymidylic acid through thymidylate synthase. 5FU, along with other antitumor chemotherapeutics, has several side effects, such as cutaneous and mucosal changes, as well as gastrointestinal, cardiovascular and hematopoietic dysfunctions. These effects require 3–4-week intervals between applications to enable detoxification through the hepatic route and excretion through the urine [7,8,9]. This time-interval between applications allows the adaptation and replication of chemoresistant variants and favors relapses [10,11]. Therefore, it is necessary developing new strategies to reduce the aforementioned symptoms.

Targeting drug delivery is a promising approach focused on enhancing the delivery of conventional chemotherapeutics to cancer tissues in order to decrease their concentration in patients’ bloodstream, and the consequent side effects of them, as well as to improve treatment efficiency. AuNPs are subjects of intense investigations in targeting therapies due to their relative safety, stability and easy preparation [12]. AuNPs’ desirable physicochemical properties comprise very small size, easy surface functionalization, as well as electrical and optical effects. Moreover, they allow surface functionalization based on several molecule types such as drugs, proteins and nucleic acids [13,14,15,16,17].

Since CRC overexpresses epidermal growth factor receptors (EGFR)-transmembrane glycoproteins belonging to the tyrosine kinase receptor family [18], the aim of the current study was to enhance the delivery of 5-FU antineoplastic agents carried by AuNPs to target cells by coating them with anti-EGFR monoclonal drug antibodies. Results have shown that some compounds synthesized by our research group have induced apoptosis/necrosis and impaired the proliferation of colon cancer cell lines ‘HCT-116′ and ‘HT-29′.

## 2. Materials and Methods

### 2.1. Synthesis of Gold Nanoparticles and Surface Functionalization Based on 11-Mercaptoundecanoic Acid

AuNPs were synthesized through the conventional method based on tetrachloroauric acid (HAuCl_4_—Sigma-Aldrich, São Paul, Brazil) reduction by using sodium citrate (Sigma-Aldrich) [19,20]. Briefly, a 0.1 M HAuCl_4_ solution was boiled in reflux chamber under constant stirring and added to a 0.68 M di-hydrated sodium citrate solution. The mixture was kept under ebullition and stirring for approximately 5 min, when the dark solution changed into a red-wine shade [19]

AuNP surface was modified by the treatment with ethanolic solution composed of 11-mercaptoundecanoic acid (MUA-Sigma-Aldrich) at 0.1 mg/mL in conical assay tube, which enabled thiol group deposition on gold surface. MUA incorporation was evaluated based on visible light spectroscopy.

### 2.2. Carboxyl Modification by EDC/NHS

Free carboxyl endings of MUA were treated with *N*-(3-Dimethylaminopropyl)-N′-ethylcarbodiimide (EDC—Sigma-Aldrich) in association with *N*-hydroxysuccinimide (NHS—Sigma-Aldrich)-in other words, a 0.4 M EDC solution was used in association with a 0.1 M NHS solution (at 1:1 ratio) [19]. One microliter of MUA-modified AuNP was added with 100 µL of EDC/NHS solution; free carboxyl modification was evaluated based on visible light spectroscopy.

### 2.3. Fluorouracil and Anti-EGFR Incorporation

Compounds were prepared by mixing the MUA-EDC/NHS-modified AuNP suspension with 7.7 M 5-FU and 100 µg/mL of anti-EGFR antibody (5000 μg/mL). The mixture was stirred at 50 rpm overnight, at 2–8 °C; next, it was centrifuged at 8.000× *g* for 5 min. The supernatant was discarded and the compound was washed twice in 1% sodium citrate [21,22]. Finally, nanoparticles were suspended in 10 mL of 1% sodium citrate and sterilized through their exposure to UV light for 3 h. Sterilization was investigated by culturing AuNP samples in RPMI 1640 culture medium supplemented with fetal calf serum (FCS), 1% nonessential amino acids, 1% sodium pyruvate and 25 uM HEPES (complete culture medium) at 37 °C and 5% constant CO_2_ tension for 48 h. Nanoparticle suspensions were named AuNP, AuNP 5FU, AuNP EGFR and AuNP 5FU EGFR; they were kept at 4 °C for 30 days, at most.

### 2.4. Transmission Electron Microscopy

The ultrastructure of synthesized compounds was analyzed based on transmission electron microscopy (TEM) in order to characterize changes induced by ligand incorporation. Samples were drop coated on 150-mesh copper supports and analyzed in Tecnai G2 Spirit BioTWIN microscope (FEI Company) at the Laboratory of Structural Characterization of the Material Engineering Department, Federal University of São Carlos (UFSCar), SP, Brazil. The equipment was set based on the following parameters: opening C2 = 2; acceleration voltage: 200 kV; spot-scan 8, magnitude: 160 Kx, 620 Kx and 1.25 Kx.

### 2.5. Zeta Potential (ζ)

AuNP compounds were added to “Y”-shaped acrylic cuvettes equipped with electrodes to enable analyzing the hydrodynamic Zeta (ζ) potential. Measurements were taken at the Laboratory of Electrochemistry and Laboratory of Nanomedicine and Nanotoxicology, Chemistry Institute of São Carlos (IQSC), Physics Institute (IFSC) of University of São Paulo (USP).

### 2.6. Calculating the Number of Particles

The number of particles in different suspensions was calculated based on particles’ size, by using the Haiss’ formula [23]:

(1) $$N=\frac{A_{450}\times 10^{14}}{d^{2}[-0.295+1.36\exp\left(-{\left(\frac{d-96.8}{78.2}\right)}^{2}\right){]}^{}},$$

where in:

*N* = number of particles/mL;
*A*_450_ = absorbance under 450 nm;
*d* = particles’ diameter (nm).

### 2.7. Colorectal Cancer Cell Lines

Human colorectal cancer cells HCT-116 (resistant to anti-EGFR) were collected at the Cell Bank of Rio de Janeiro, whereas HT-29 (low sensitiveness to anti-EGFR) was gently provided by Dr. Rodrigo Hernandes from the Microbiology and Immunology Department, Institute of Biosciences, UNESP. These cells were cultured in 75 cm^2^ -culture flasks filled with complete culture medium at 37 °C under 5% constant CO_2_ tension, until reaching 80% confluence. The use of these cells in the present study was approved by the Ethics Committee of Botucatu Medical School (CEP-FMB), UNESP-SP-Brazil, according to certification n. 71397817.6.0000.5411.

### 2.8. Challenging Tumor Cells In Vitro with AuNP Compounds

The antitumor activity of AuNP compounds was evaluated by exposing tumor cells in vitro to 10^10^ or 2 × 10^10^ nanoparticles/mL. Approximately 5 × 10^4^ cells were cultured in 24-well culture plate and kept overnight at 37 °C and 5% CO_2_ to enable cell adherence to the surface (1 mL). Next, 2 × 10^10^ of AuNP were transferred to the wells and cultured for 24 h or 48 h. Besides each compound (AuNP, AuNP 5FU, AuNP EGFR and AuNP 5FU EGFR), the current study also used 0.38 mM of pure 5FU solution as positive control. Cells were detached by using 200 µL of trypsin (3 min at 37 °C) and culture wells were washed in 500 µL of complete culture medium for further cell death analysis.

### 2.9. Tumor Cell Death

Cells challenged with AuNP compounds were removed from the culture plates, transferred to assay tubes and washed twice in annexin V buffer solution at 1200 rpm for 8 min. Next, they were labeled by annexin V-PE and 7-adenosine-actinomycin D-PerCP (7-AAD; BD Biosciences). Annexin V bound to phosphatidylserine. Healthy cells kept phosphatidylserine in the inner surface of plasma membrane, while cells under apoptosis externalized this phospholipid and were labeled by this fluorescent reagent (which defines early apoptosis). On the other hand, 7-AAD bound to chromosomes by DNA intercalation. Dead cells lost plasma membrane integrity and allowed 7-AAD to reach the chromosomes, as well as DNA labeling. Cells labeled by annexin-V and 7-AAD have indicated cell death by apoptosis (cells under late apoptosis). These phenomena were analyzed through flow cytometry in FACS Canto^TM^ II (BD Biosciences) in the FACSDiva^TM^ software. Results were analyzed in the FlowJo software, version vX.10.6 (Tree Stars Inc.) [24]; they were normalized by the control and expressed as toxicity index, which was calculated through the formula presented below:

(2) $$Toxicity\;index=\;\frac{\%\;nanoparticle-induced\;apoptosis}{\%\;basal\;apoptosis}.$$

### 2.10. Cell Proliferation Assay

Cell proliferation was evaluated after the treatment with AuNP compounds was concluded. Cells were labeled with CellTrace™ far Red-APC (ThermoFisher Scientific, Rio de Janeiro, Brazil) before treatment, according to the manufacturer’s instructions. Briefly, 10^6^ cells were placed in conical tube, centrifuged, suspended in 1 mL of phosphate buffer solution (PBS) and added with 0.25 µL of CellTrace™. Next, they were incubated in dark box for 20 min, added with 5 mL of complete culture medium and incubated for 5 min. Subsequently, cells were washed and cultured in 96-flat bottomed well culture plates, along with AuNP compounds, for 96 h. Next, they were detached and analyzed based on flow cytometry, as previously described. This assay was based on labeling cell cytoplasm with fluorescent reagent (CellTrace™) before it was exposed to the nanoparticles. Living cells were permeable to CellTrace, a fact that enabled fluorescent DNA labeling. Fluorescent DNA signal has decreased due to cell replication; thus, cell division calculation was inversely proportional to fluorescent intensity.

### 2.11. Statistical Analysis

All experiments were run in triplicate and results were expressed as mean ± SD. Data were subjected to Bartlett’s test for homogeneity analysis. Parametric data were subjected to ANOVA followed by the Tukey or Dunnett’s test. Non-parametric data were subjected to the Kruskal–Wallis test. Differences were significant at error probability lower than 5% (*p* ≤ 0.05).

## 3. Results

### 3.1. Physicochemical Characterization of AuNP

Table 1 shows AuNP dimensions calculated based on images generated through transmission microscopy in the Image J software. There were particles smaller than 20 nm. Pure particles measured approximately 11.5 nm, as expected for the synthesis technique used in the current study. Subsequent incorporations have slightly increased their size, mainly in AuNP 5FU EGFR, whose particles were 8 nm bigger than the ones observed in pure AuNP. Zeta potential (Table 1) analysis has shown negative charge profile in pure AuNP and in the ones carrying chemotherapeutic agents. Nanoparticles carrying just antibodies have shown charge close to zero. These results have indicated good independence between nanoparticles’ size and polydispersity, whereas the negative zeta potential value has given stability to AuNPs and prevented their aggregation.

**Figure 1:**
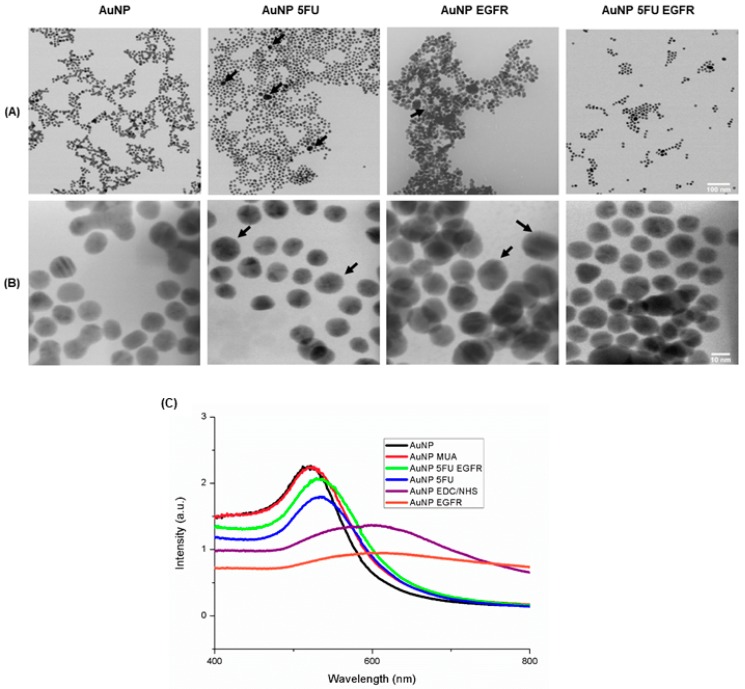
Physicochemical characterization of AuNP and constructs by transmission electron microscopy (TEM) of AuNP. The scales are in the sizes of (**A**) 100 nm and (**B**) 10 nm, respectively of AuNP; AuNP 5FU; AuNP EGFR and AuNP 5FU EGFR. (**C**) UV-vis spectra: on black line AuNP without modification; on pink line, modified with MUA; on green line modified with 5FU EGFR; on blue line AuNP 5FU; on purple line EDC/NHS and on orange line EGFR.

The calculation method described by Haiss, which is based on particles’ size and wavelength [23] and on estimated amounts of 0.5 to 2.9 × 10^12^ nanoparticles/mL, was used to determine the number of particles used in assays conducted in vitro (Table 1). Figure 1C shows the shift in absorbance picks and the enhanced wavelength observed during UV-vis spectroscopy, which together indicate molecule incorporation to AuNP surface. The two curves (AuNP and AuNP MUA, 518 and 519, respectively) were very similar; however, the peak moved to the right after EDC/NHS (591 nm) addition, a fact that indicated particle aggregation—as evidenced in the color change from red wine to purple. 5FU addition has reset the peak to the initial range of 534 nm, and the color shifted back to red wine in compliance with a previous report [25]. Anti-EGFR antibody addition has moved the peak to the right (605 nm) and induced significant intensity decrease. AuNP loaded with 5-FU and EGFR (531 nm), has shown a peak similar to that of AuNP 5-FU, although with higher intensity. Thus, it is possible monitoring the growth of AuNPs along with the functionalization of the surface, so that the color shift from red to purple could be attributed to increased AuNP size after ligand adsorption, as well as to electric dipole–dipole interaction and coupling between adjacent particles that make their agglomeration easier.

The AuNP ultrastructural analysis based on TEM has shown the morphology of pure AuNPs and all functionalized variations of it. Images were taken at scales 100 nm (Figure 1A) and 10 nm (Figure 1B) and they showed spherical-shaped 11–18-nm polydisperse particles, except for AuNP/EGFR, which showed agglomeration. AuNP/5FU/anti-EGFR particles were slightly larger than the other ones; this outcome is in compliance with data collected through UV-vis spectroscopy.

### 3.2. AuNP 5FU EGFR Induces Apoptosis in Colorectal Tumor Cells

The toxicity of AuNPs to HCT-116 and HT-29 colorectal cancer cell lines was analyzed after the characterization procedure was over. AuNPs were placed on cell monolayers and incubated for 24 h or 48 h to enable analyzing their ability to induce apoptosis and cell death. The flow cytometry analysis has shown that cells undergoing early apoptosis were labeled with annexin V at quadrant Q1, necrotic cells were labeled with 7-AAD at quadrant Q3 and double-positive dead cells underwent late apoptosis at quadrant Q2.

**Figure 2:**
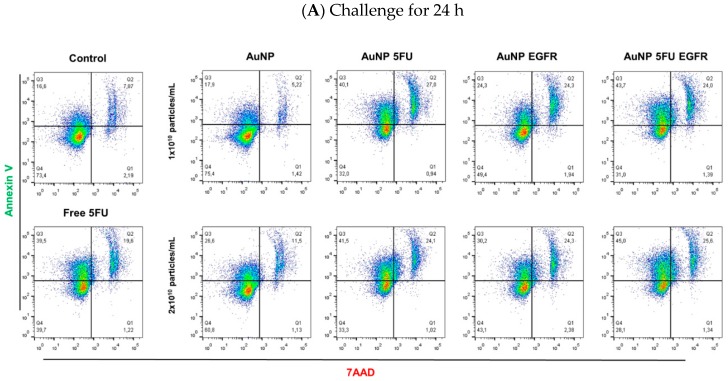

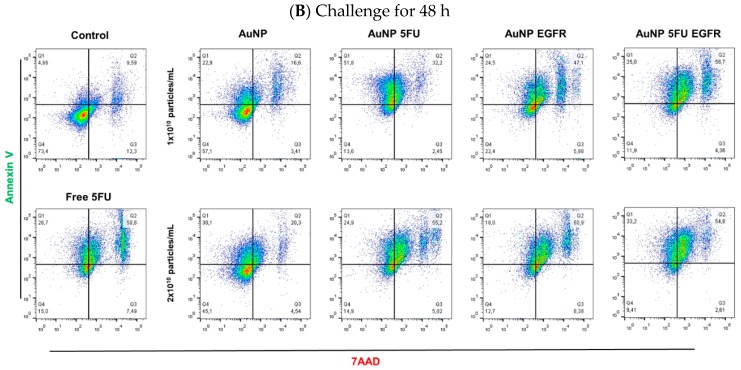
Representative assay of apoptosis and necrosis of HT-29 cells induced by free 5FU, AuNP, AuNP 5FU, AuNP EGFR or AuNP 5FU EGFR at the concentrations of 10^10^ and 2 × 10^10^ particles/m, for 24 h (**A**) or 48 h. (**B**). Our analysis by flow cytometry shows the efficiency of the AuNP 5FU EGFR and AuNP EGFR to increase the number of cells under late apoptosis with less than 10% viable cells at the highest concentration tested (representative data of three replicates).

**Figure 3:**
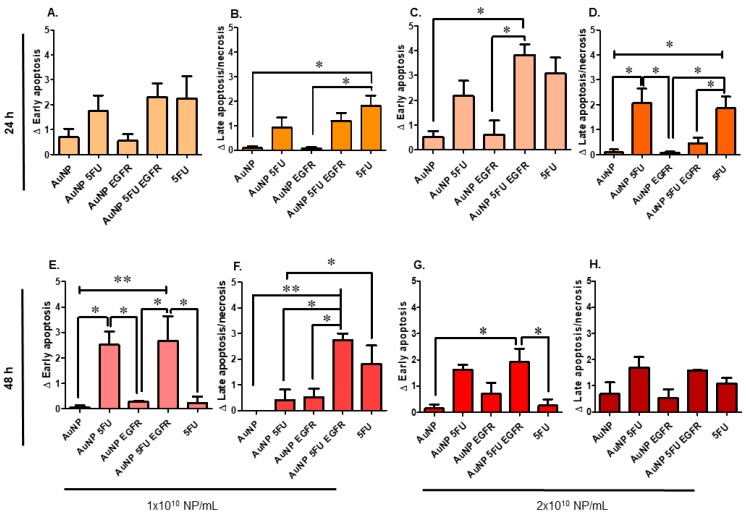
Cytotoxicity of the different concentrations of composites on HT-29 cells treated for 24 and 48 h. In the upper left, show the treatments with 1 × 10^10^ nanoparticles/mL in 24 h: (**A**). Early apoptosis (annexin V^+^); (**B**). late apoptosis/necrosis (annexin V^+^ and 7AAD^+^) and in the upper right, show the treatments with 2 × 10^10^ nanoparticles/mL in 24 h: (**C**). early apoptosis; (**D**). late apoptosis/necrosis. In the lower left, show the treatments with 1 × 10^10^ nanoparticles/mL in 24 h: (**E**). early apoptosis; (**F**). late apoptosis/necrosis and in the lower right, show the treatments with 2 × 10^10^ nanoparticles/mL in 24 h: (**G**). early apoptosis and (**H**). late apoptosis/necrosis. Statistical analysis was performed by one-way ANOVA with Tukey post-test, with significance of * *p* < 0.05 and ** *p* < 0.001.

Figure 2 shows dot plots of HT-29 cells challenged by nanoparticles for 24 h (Figure 2A) or 48 h (Figure 2B). These figures show the efficiency of compounds carrying anti-EGFR in comparison to pure AuNP, AuNP 5FU and pure 5FU (0.38 mM). The larger the number of nanoparticles added to the assay, the higher the apoptosis levels (Figure 3). Results have shown that HT-29 cells were induced to early apoptosis after they were exposed to 2 × 10^10^ of AuNP 5FU EGFR for 24 h (Figure 3 upper), whereas 10^10^ and 2 × 10^10^ were active under 48-h challenge (Figure 3 lower). It is important emphasizing that the pro-apoptotic effect of compounds carrying 5FU and anti-EGFR was higher than that of the drug used in separate after 48 h (Figure 3E,F); on the other hand, pure AuNP had just minimal effect on cell viability.

**Figure 4:**
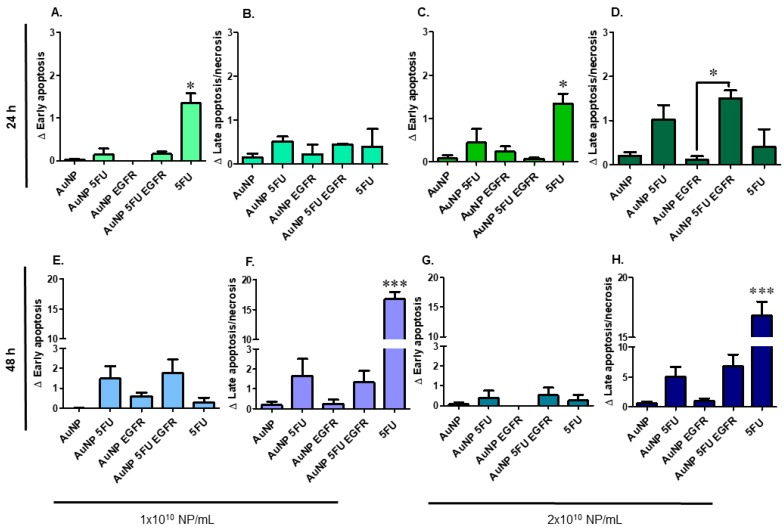
Cytotoxicity of gold nanoparticles and functionalizations in different concentrations on HCT-116 human colorectal carcinoma cells for 24 and 48 h. In the upper left, show the treatments with 1 × 10^10^ nanoparticles/mL in 24 h: (**A**). cells in recent apoptosis (annexin V^+^); (**B**). late apoptosis/necrosis (annexin V^+^ and 7AAD^+^) and in the upper right, show the treatments with 2 × 10^10^ nanoparticles/mL in 24 h: (**C**). cells in recent apoptosis and (**D**). late apoptosis/necrosis. In the lower left, show the treatments with 1 × 10^10^ nanoparticles/mL in 24 h: (**E**). cells in recent apoptosis and (**F**). late apoptosis/necrosis. In the lower right, show the treatments with 2 × 10^10^ nanoparticles/mL in 24 h: (**G**). cells in recent apoptosis and (**H**). late apoptosis/necrosis. Statistical analysis was performed by one-way ANOVA with Tukey post-test, with significance of * *p* < 0.05 and *** *p* < 0.0001.

HCT-116 cells were more resistant than HT-29, since they showed lower apoptosis levels after being challenged by the compounds for 24 h (Figure 4 upper). It was possible observing slight AuNP 5FU EGFR ability to promote early apoptosis in assays using 10^10^ and 2 × 10^10^ particles/mL (Figure 4A,C), only after 48 h (Figure 4 lower). Neither pure AuNP nor AuNP EGFR enabled apoptosis and/or necrosis in these cell lines, a fact that evidenced the effectiveness of 5-FU.

### 3.3. Nanoparticles Carrying 5FU Have an Antiproliferative Effect

**Figure 5:**
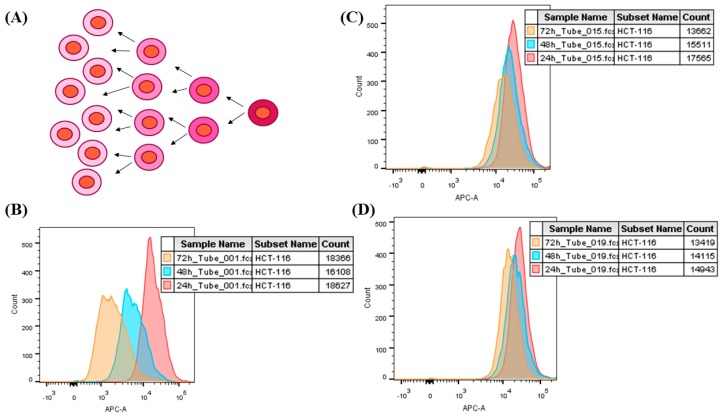
Representative figure of the cell proliferation assay by CellTrace ™. (**A**) Cells are pre-stained with CellTrace™, in the process of cell division, the dye is dividing between the cytoplasm of the daughter cells, decreasing the concentration at each cycle. (**B**) Histogram of CellTrace labeling in the control group. The red peak refers to 24 h of treatment; or blue 48 h; or orange 72 h. Each peak represents the cell division and consequently dilution of CellTrace into the cytoplasm. (**C**) Treatment with AuNP 5FU EGFR at the concentration of 2 × 10^10^ NP/mL. Cells from this treatment did not proliferate due to cytotoxicity of the treatment, also noted the decrease in fluorescence intensity. (**D**) Treatment with free 5FU (0.38 mM), in this treatment the cells did not suffer cell division due to their cytotoxic action.

**Figure 6:**
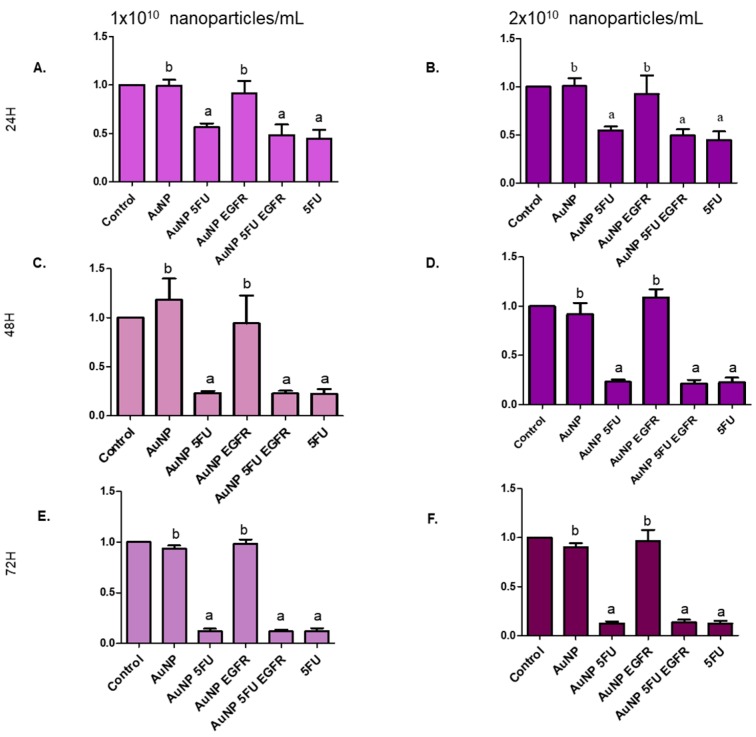
Cell proliferation index in the HT-29 colorectal adenocarcinoma line evaluating the dilution of CellTrace™ into the cytoplasm of cells. The cells were stained, treated (1 or 2 × 10^10^ NP/mL) and collected at 24, 48 and 72 h: (**A**) 24 h with 1 × 10^10^ NP/mL; (**B**) 24 h with 2 × 10^10^ NP/mL; (**C**) 48 h with 1 × 10^10^ NP/mL; (**D**) 48 h with 2 × 10^10^ NP/mL; (**E**) 72 h with 1 × 10^10^ NP/mL and (**F**) 72 h with 2 × 10^10^ NP/mL. Statistical analysis was performed by one-way ANOVA followed by Dunnett’s post-test, with significance of *p* < 0.05.

**Figure 7:**
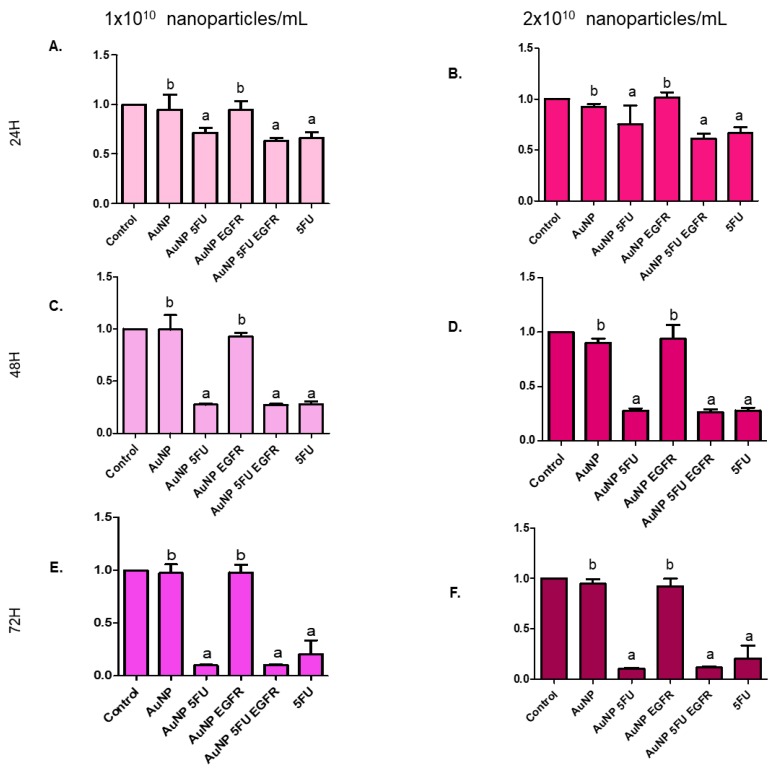
Cell proliferation index in the HCT-116 colorectal carcinoma line evaluating the dilution of CellTrace™ into the cytoplasm of cells. The cells were stained, treated (1 or 2 × 10^10^ NP/mL) and collected at 24, 48 and 72 h: (**A**) 24 h with 1 × 10^10^ NP/mL; (**B**) 24 h with 2 × 10^10^ NP/mL; (**C**) 48 h with 1 × 10^10^ NP/mL; (**D**) 48 h with 2 × 10^10^ NP/mL; (**E**) 72 h with 1 × 10^10^ NP/mL and (**F**) 72 h with 2 × 10^10^ NP/mL. Statistical analysis was performed by one-way ANOVA followed by Dunnett’s post-test, with significance of a < b = *p* < 0.05.

Evidences of the antitumor role played by the herein tested compounds were reinforced by their ability to block the proliferation of tumor cells. Figure 5 shows that compounds carrying 5FU were more efficient in inhibiting the proliferation of HT-29 (Figure 6) and HCT-116 cells (Figure 7) than compounds carrying only anti-EGFR antibody or pure AuNP. The aforementioned cells were equally affected by the exposure to the tested compounds, mainly to AuNP 5FU EGFR, in all treatment periods (24, 48 and 72 h), with emphasis on the 72 h period. This antiproliferative effect was not dose-dependent, since both 10^10^ and 2 × 10^10^ particles/mL have shown the same ability to affect cell proliferation.

## 4. Discussion

Several groups have reported the use of AuNPs to deliver drugs to tumor cells, but the combination of chemotherapeutics and antibodies in a single small particle is a significant challenge due to a range of chemical interactions between gold and antibodies [26]. The present study has produced and characterized AuNPs smaller than 20 nm, which were capable of carrying 5-FU to colorectal cancer cells overexpressing EGFR.

Gold nanoparticles coupled to 5FU and anti-EGFR antibody have significantly affected the viability of HT-29 tumor cells capable of inducing apoptosis and necrosis in a time-dependent fashion. In addition, the proliferation of both HT-29 and HCT-116 was affected regardless of the nanoparticle concentrations cells were exposed to. Thus, analyzed data have indicated that the biological effects of AuNPs have improved due to the combination of chemo- and immunotherapeutic agents to be carried by nanoparticles.

Nanoparticles observed in TEM presented diameter of approximately 10–15 nm; no significant change was induced by 5FU incorporation, since these molecules have 5.3 Å [27]. Such observation was similar to the report claiming that AuNPs coupled to glutathione [25] or cisplatin also kept the same size [28]. Therefore, UV-vis spectroscopy was used to evaluate the incorporation of new material to AuNP [29]; such incorporation was evidenced by the dislocation of peaks to the right, which has indicated increased absorbance due to such material. In addition, some suspensions have shown small aggregates; it may have happened mainly due to antibody incorporations that have induced non-covalent links and enabled the approximation of these particles [26].

The current results were completed by zeta potential analysis-this potential keeps AuNP dispersity because surface charge higher than −20 mV repels the particles and sustains dispersity. On the other hand, the combination between AuNP and antibodies alone presents surface charge close to zero, a fact that can explain the easy aggregation [30]. According to Deka et al., a single-stranded DNA can sequester citrate AuNP ions and make aggregation easier; assumingly, antibodies can do it as well [22].

The antitumor potential of AuNP compounds against colon cancer cell lines was analyzed after the physicochemical characterization was concluded. Cells were challenged for 24 h and 48 h based on the assumption that 5FU would be slowly released after the interaction between nanoparticles and target cells, as previously reported [19]. The first relevant observation made in the current study was that pure AuNPs did not show direct toxicity to target cells, whereas 5FU incorporation made them able to induce apoptosis and inhibited the proliferative ability of tumor cells. A similar profile was reported by Safwat et al. [25], who synthesized AuNPs functionalized with glutathione and 5-FU to kill colorectal cancer cells in an assay conducted ex vivo. According to another study, AuNP 5FU has shown antitumor effect 20% higher than that of pure 5-FU at 1 × 10^−4^ M, whereas pure nanoparticles were inert [31]. Therefore, the current results appear to be more promising than the ones reported in other studies, since it evidenced the direct non-specific toxicity of gold nanoparticles [32].

Cytotoxic effect has improved due to 5FU and anti-EGFR incorporation to AuNPs. The early apoptosis of colon cancer cell lines was mainly affected by the highest concentration, with emphasis on HT-29 cells. Such effect was even higher than that of pure 5FU. This difference may have happened due to significantly higher EGFR expression on the surface of HT-29 cells than on the surface of HCT-116 cells. In fact, HT-29 cells are poorly sensitive to anti-EGFR, whereas HCT-116 cells are resistant to this antibody, a fact that reinforces the specificity and efficiency of the selected antibody to drive particles towards target cells [33], as well as the positive effect of it conjugation on AuNP.

Cetuximab was the commercially available clinical preparation used in the current study. It is a chimeric monoclonal IgG directed to EGFR; it is prepared in excipient capable of maintaining antibody stability. This excipient can interfere in colloid formation after antibodies’ incorporation to AuNPs, a fact that turns such incorporation into a delicate procedure, since they sequester citrate ions capable of maintaining stability and of avoiding aggregation [22]. Sodium citrate was added during the wash steps in order to prevent massive reaction to ions in the particles, as well as to reduce the risk of aggregation. However, despite the efforts made in the current study, AuNPs containing only antibodies remained aggregated, a fact that may explain the fact that AuNP EGFR did not produce any biological effect on the herein conducted tests.

## 5. Conclusions

The combination of 5-FU and anti-EFGR loading in the gold carrier has successfully stopped cell proliferation; this effect may be due to its ability to induce apoptosis in target cells. The cytotoxicity analysis has evidenced the superior efficiency of this methodology in inducing apoptosis in both systems in comparison to the drug-free methodology. On the other hand, pure AuNPs did not present direct cytotoxic effects, which is desirable in nanostructure-based therapeutic proposals. Based on the current data, 5-FU entrapped AuNP can open new nanomedicine perspectives and enable the development of new therapies focused on targeting cells that overexpress EGFR.

## Figures and Tables

**Table 1:** Physicochemical characterization of AuNP and their constructs.

Formulation *	Diameter (nm)	Zeta Potential (mV)	Radius (nm)	A_450_	N° of Particles (×10^12^) mL^−1^
AuNP	11.5 ± 1.06	−46.5 ± 1.10	5.75	1.556	2.95
AuNP 5FU	12.8 ± 1.5	−44.4 ± 2.90	6.4	1.150	1.73
AuNP EGFR	14.9 ± 1.23	−7.45 ± 0.131	7.45	0.71T6	0.843
AuNP 5FU EGFR	18.83 ± 1.52	−33.1 ± 3.78	9.415	1.316	1.186

Physicochemical characterization of AuNP*. Size calculation was based on TEM images (Figure 1b.) measured with ImageJ software; zeta potential were Zetasizer Nano ZS90; we also estimated number of particles/mL using the particle diameter obtained by ImageJ and absorbance at 450 nm (A_450_) and mass using the radius and number of particles, both obtained by the Haiss formula. * AuNP: nanoparticles without functionalization; AuNP-5FU: AuNP functionalized with 5FU; AuNP-EGFR: AuNP functionalized with antibody anti-EGFR. AuNP-5FU EGFR: AuNP functionalized with 5FU and anti-EGFR antibody.
